# Spatio-temporal modelling of referrals to outpatient respiratory clinics in the integrated care system of the Morecambe Bay area, England

**DOI:** 10.1186/s12913-024-10716-7

**Published:** 2024-02-22

**Authors:** Rachael Mountain, Jo Knight, Kelly Heys, Emanuele Giorgi, Timothy Gatheral

**Affiliations:** 1 Lancaster Medical School, Lancaster University, Lancaster, UK; 2 University Hospitals of Morecambe Bay NHS Foundation Trust, Westmorland General Hospital, Kendal, UK

**Keywords:** Chronic respiratory disease, Integrated care, Outpatient referrals, Routinely collected data, Spatio-temporal

## Abstract

**Background:**

Promoting integrated care is a key goal of the NHS Long Term Plan to improve population respiratory health, yet there is limited data-driven evidence of its effectiveness. The Morecambe Bay Respiratory Network is an integrated care initiative operating in the North-West of England since 2017. A key target area has been reducing referrals to outpatient respiratory clinics by upskilling primary care teams. This study aims to explore space-time patterns in referrals from general practice in the Morecambe Bay area to evaluate the impact of the initiative.

**Methods:**

Data on referrals to outpatient clinics and chronic respiratory disease patient counts between 2012-2020 were obtained from the Morecambe Bay Community Data Warehouse, a large store of routinely collected healthcare data. For analysis, the data is aggregated by year and small area geography. The methodology comprises of two parts. The first explores the issues that can arise when using routinely collected primary care data for space-time analysis and applies spatio-temporal conditional autoregressive modelling to adjust for data complexities. The second part models the rate of outpatient referral via a Poisson generalised linear mixed model that adjusts for changes in demographic factors and number of respiratory disease patients.

**Results:**

The first year of the Morecambe Bay Respiratory Network was not associated with a significant difference in referral rate. However, the second and third years saw significant reductions in areas that had received intervention, with full intervention associated with a 31.8% (95% CI 17.0-43.9) and 40.5% (95% CI 27.5-50.9) decrease in referral rate in 2018 and 2019, respectively.

**Conclusions:**

Routinely collected data can be used to robustly evaluate key outcome measures of integrated care. The results demonstrate that effective integrated care has real potential to ease the burden on respiratory outpatient services by reducing the need for an onward referral. This is of great relevance given the current pressure on outpatient services globally, particularly long waiting lists following the COVID-19 pandemic and the need for more innovative models of care.

**Supplementary Information:**

The online version contains supplementary material available at 10.1186/s12913-024-10716-7.

## Background

Chronic respiratory disease (CRD) remains a leading cause of morbidity and mortality in the UK; it is estimated that 15% of the population have a history of CRD and it is the fourth most common cause of death in England [1, 2]. Respiratory disease disproportionately affects disadvantaged socio-economic groups due to the known links with risk factors such as smoking, air pollution, poor housing, and occupational hazards [3]. CRD represents a large burden on the NHS with estimated direct costs of £4.7 billion from asthma and chronic obstructive pulmonary disease (COPD) alone [4]. The pressure is set to increase with an ageing population [5] which raises questions about how respiratory services can be changed to be more efficient and provide the best possible care for patients.

Promoting integrated care is a key goal of the NHS Long Term Plan to improve population respiratory health [6]. Integrated care is an organising principle for care delivery that seeks to improve the quality of care for patients by providing services that are better coordinated and act in a joined-up way [7, 8]. Integrated care has been argued as the key to making the health and social care system more sustainable. Without integration patients are more likely to become lost in the system, needed services can be duplicated or delayed, and the potential for cost-effectiveness declines [9]. However, despite the large push toward building integrated systems of care across England in recent years, evaluations have historically produced mixed results [10–12]. Research suggests this could, at least partly, be caused by the challenge in selecting outcome measures that are able to quantify the success of complex and multi-faceted initiatives [10, 13, 14]. The issue is exacerbated by data access barriers, particularly access to data linked across healthcare tiers at patient level, that can limit the possibilities for evaluations [14, 15].

The North-West region has the highest under 75 mortality rate from respiratory disease in England, 44.7% compared to 33.6% country-wide [16]. Clinical commissioning groups were dissolved on $1^{\text {st}}$ July 2022, but at the time of this analysis, they were NHS bodies responsible for the planning and commissioning of healthcare services for their local area in England. The Morecambe Bay Clinical Commissioning Group (MBCCG) in the North-West of England provided primary care for approximately 352,000 patients across 32 general practices (GPs) [17]. The majority of patients reside in Lancaster, South Lakeland, and Barrow-in-Furness, covering both rural and urban town areas, as well as a range of socio-economic levels including some of the most deprived communities in the country [18].

**Fig. 1:**
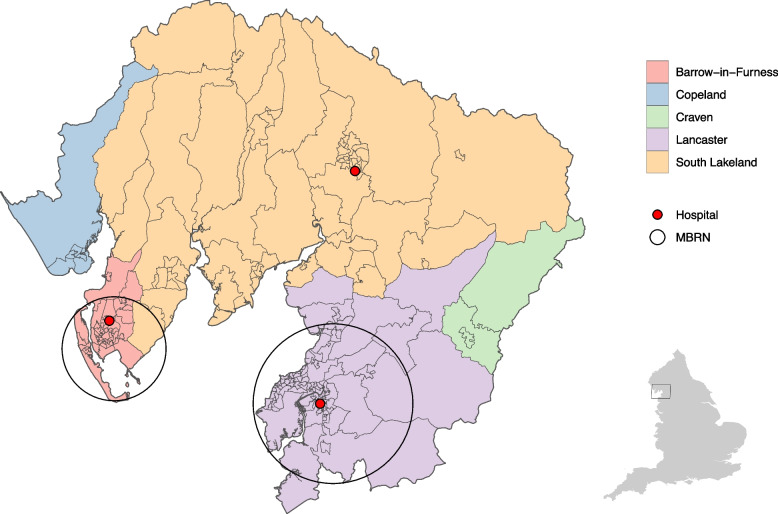
Map of Morecambe Bay area shaded by local authority. Black circles show the approximate area of influence of the Morecambe Bay Respiratory Network

The Morecambe Bay Respiratory Network (MBRN) is an integrated care initiative operating in the Morecambe Bay area that aims to improve the quality and efficiency of healthcare delivery for patients with the four most prevalent CRDs in the UK: asthma, COPD, bronchiectasis, and interstitial lung disease (ILD). The first phase of the initiative began in 2017, reaching 50% of the MBCCG population through 8 practices in the network, clustered predominately in the Lancaster and Barrow-in-Furness localities (Fig. 1). A second phase in 2019 extended that reach to 65%, but this research focuses on phase one.

The MBRN evolved out of the vanguard programme for new care models, receiving approximately £1 million investment (£3 per patient per practice) from the MBCCG to develop a model of care that effectively used existing services to produce efficient outcomes [19, 20]. Given NHS consultant recruitment challenges in the area and nationally, models creating bespoke new services were unsuitable [20, 21] The core components of the MBRN model include an enhanced primary care team that has direct access to specialist investigation and is closely supported by secondary care expertise via monthly multidisciplinary meetings. This contrasts with other integrated respiratory initiatives, such as Knowsley Community Respiratory Service [22], also in the North-West region, that has moved acute services to the community rather than empowering primary care to provide a higher level of clinical care. The MBRN promotes effective communication and shared pathways across healthcare tiers to ensure that patients receive consistent information and to remove unnecessary or duplicate appointments. A key metric for the MBRN has been the measured impact on outpatient referrals reflecting the goals of improved service efficiency, bringing care closer to the patient, and avoiding unnecessary referrals that increases pressure on outpatient services and wait time for all patients.

NHS England undertakes 125 million outpatient appointments a year [23]. The COVID-19 pandemic has added considerable pressure to an already strained system with 6 million people on the waiting list for elective care compared to 4.4 million prior to the pandemic. The waiting list is expected to continue to grow in the short term as patients come forward who have delayed seeking health advice or treatment during the pandemic [24]. The radical redesign of elective care is more essential now than ever to manage demand in a way that improves patient care as well as service efficiency [25]. There is a need to work with primary care to improve patient pathways to reduce the need for an onward referral and avoidable delays where possible [24].

The aim of this research is to provide a data-driven assessment of the impact of the MBRN using a source of routinely collected data that has not been extensively used in health service research. This analysis focuses on referrals to outpatient respiratory clinics, an outcome measure of key relevance both to the MBRN and wider NHS agenda. Existing quantitative evaluations of integrated care initiatives for respiratory disease often focus on hospital utilisation in terms of non-elective admissions, with mixed results [22, 26–28]. The literature on the impact to outpatient referrals is lacking. Evaluations for non-respiratory primary care enhanced initiatives have found evidence of a reduction in outpatient referrals, but these studies were restricted to short time frames (3-6 months) and did not account for other factors [29, 30]. The use of routinely collected data in this analysis facilitates a modelling approach that adjusts for demographic factors and changes in CRD patient count to closer study the underlying referral behaviour.

The remainder of the paper is structured as follows. After a brief overview of the modelling approach, we describe the routinely collected data source, including complexities and sources of missingness and the impact this may have on space-time analyses at small-area geography level. Next, we propose the methodology used that has two parts:

1. Spatio-temporal extension of conditional autoregressive models to adjust for the complexities in the data prior to the primary analysis.
2. Generalised linear mixed model of outpatient referrals in the Morecambe Bay area over an eight year period.

We then present the results of the model output before providing a concluding discussion, relating back to the impact of the MBRN, the wider context of the demand on outpatient services, and the importance of robust data for healthcare evaluations.

## Methods

The main outcome variable is annual rate of referrals from GP to outpatient respiratory clinics over an eight year study period ($1^{\text {st}}$ April 2012 - $31^{\text {st}}$ March 2020) for 204 of the Lower-layer Super Output Areas (LSOAs) that lie within the MBCCG boundaries. LSOAs are small areas used for census geography in the UK that have an average population size of 1,500 [31]. The rate denominator of the outcome measure is number of diagnosed CRD patients to adjust for differences in patient count over space and time, and to avoid a model where referrals is acting as a proxy for prevalence. We consider data from adults aged 25 years or over. The 18-24 age bracket was excluded to reduce potential bias from the large student population in central Lancaster. Further, two LSOAs within the MBCCG boundaries were excluded due to the influence of Lancaster University.

For the sake of brevity, in the remainder of the paper study years will be referenced by the start date. For example, the study year “2012” will refer to the period $1^{\text {st}}$ April 2012 - $31^{\text {st}}$ March 2013. Additionally, “adults” will refer to individuals aged 25 years or over unless specified otherwise.

### Primary data source

This study uses routinely collected NHS data stored in the Morecambe Bay Community Data Warehouse (CDW), a SQL Server owned and maintained by the University Hospitals of Morecambe Bay NHS Foundation Trust. The CDW contains data from primary, secondary, and community care across Morecambe Bay and uses pseudonymised NHS Numbers to allow linkage between data sets at an individual level.

Referrals were identified from secondary care records of the three hospitals within the study area with outpatient respiratory services: Furness General Hospital in Barrow-in-Furness, Royal Lancaster Infirmary in Lancaster, and Westmorland General Hospital in South Lakeland (Fig. 1). A relevant referral was defined as any new referral from GP to a respiratory, spirometry, oxygen, or lung clinic, for an adult residing in the study area. We excluded referrals to clinics for asthma biologics, respiratory postoperative, respiratory physiotherapy, sleep apnoea, and referrals made under the “two-week wait” pathway for suspected respiratory cancer. Clinics were excluded if they were outside the scope of the MBRN (e.g., cancer), had their own existing referral pathway (e.g., sleep apnoea), or if the clinic did not exist for the entirety of the study period (e.g., asthma biologics) as this may confound results.

Primary care records were used to build a GP-registered population dataset of all adults residing in a study LSOAs and registered at a MBCCG GP. An individual’s entry date is defined as the most-recent of GP registration start date and their $25^{\text {th}}$ birthday. Although registration status is recorded in the CDW, registration end date is missing for all individuals who have left or died so we use last interaction with primary care (appointment, consultation, or medication issue) as a proxy. An individual’s end date in the GP-registered population dataset, if relevant, is end date proxy or date of death.

CRD patients were identified from among the GP-registered population cohort by diagnoses recorded in primary care with a relevant asthma, COPD, bronchiectasis, or ILD SNOMED CT code. Relevant codes were identified using NHS Digital’s SNOMED CT Browser [32]. The codes were then filtered with the aim of reflecting as closely as possible MBRN’s own in-house patient registers. For an asthma diagnosis, an issuing of inhaled therapy in the past 12 months was used as an additional criterion. The Quality and Outcomes Framework guidelines [33] require post bronchodilator spirometry for a COPD diagnosis. We have not applied this criterion due to discrepancies in the recording of lung function test results in the CDW. A validation study found that using diagnoses codes alone gave a positive predictive value for true COPD of 86.5% and including spirometry results or medications only marginally improved results [34]. Start and end dates for diagnoses are recorded in the CDW and applied here to estimate the number of respiratory patients for any given space-time unit. In the case of asthma diagnoses, the ceasing of inhaled therapy for a period of 12 months qualifies as an end date.

The primary care data in the CDW has missingness and complexities that introduce bias to the GP-registered population cohort, in turn impacting the CRD patient counts. The three main issues are:

1. Two of the 32 MBCCG GPs are not signed up to the CDW data sharing agreement and so we do not have access to primary care records for these patients. This creates spatially-correlated gaps in the data.
2. We use a proxy for GP registration end date but this will likely be earlier than the true de-registration date, resulting in an underestimate of the GP-registered population size at any given time.
3. For a given registration, only a patient’s current address rather than entire address history is recorded and so movement of people within the MBCCG over time cannot be tracked. An individual’s current address is assumed to be the residency for their entire registration period which may result in individuals being assigned to an incorrect space-time unit.

Each of these issues has a spatial and/or temporal dimension and could bias the analysis via the denominator of the outcome rate.

### Secondary data sources

GP registration data from NHS Digital [35] was used to estimate the GP-registered population counts that would be observed in the CDW without the presence of bias and missingness. Since 2014, NHS Digital releases data on a quarterly basis at LSOA-level for total number of patients registered at each GP practice in England. An age breakdown is not provided at LSOA level due to possible identification of individuals when linked to other data sets. However, an age breakdown is provided for each distinct GP register. Therefore, for each of the 204 LSOAs in the study area, we estimate the number of adults registered at a MBCCG GP by multiplying the number from the LSOAs population registered at each relevant GP by the proportion of that GP’s register ages 25 years or over, then summing across all GPs. This is repeated for all quarterly releases, and the mean taken by study year.

The Office for National Statistics (ONS) publishes mid-year population estimates that are used for estimates of LSOA age and sex demographics [36]. Although the census population and GP-registered population are not identical, we use the ONS estimates since NHS Digital does not cover all study years. The MBCCG and LSOA boundaries are also available from ONS as shapefiles [37, 38]. The Open Source Routing Machine (OSRM) package in R Studio was used to construct variables for distance to healthcare services [39]. The road distance in kilometres (km) was calculated for all postcodes within the study area and then averaged by LSOA. The English Indices of Deprivation was used as a relative measure of deprivation at LSOA-level [18].

### Statistical analysis

#### Adjusting CRD patient count

We adjust the rate denominator, CRD patient count, for the previously described data complexities in the CDW GP registers by assuming that the population assigned to a given space-time unit by the CDW is representative of the corresponding true, unobserved GP-registered population. Then for study years 2014 onward, an estimate for the number of adult CRD patients is obtained for each LSOA by:

1 $$\begin{aligned} \hat{R}_{it} = \frac{R^{CDW}_{it}}{P^{CDW}_{it}} \times P^{NHS}_{it} \; , \end{aligned}$$

where $R^{CDW}_{it}$ is the CRD patient count from the CDW for LSOA *i* ($i=1,\ldots ,N$) and year *t* ($t=1,\ldots ,T$), $P^{CDW}_{it}$ the GP-registered adult population count from the CDW, and $P^{NHS}_{it}$ the GP-registered adult population estimate from NHS Digital. Since LSOA-level data is not available from NHS Digital pre-2014, we apply spatio-temporal modelling techniques to model the error in the primary care records of the CDW and to predict the NHS Digital figures for study years 2012 and 2013 based on the corresponding CDW count. Once the predictions are obtained, the adjustment in (1) can be applied.

The study period has $T=8$ years (2012-2019), but for this model we also use data from $1^{\text {st}}$ April 2020 to $31^{\text {st}}$ March 2021 to improve prediction capacity. The outcome variable is $P^{NHS}_{it}$ for LSOA *i* ($i=1,\ldots ,N$) and year *t* ($t=1,\ldots ,T+1$). The counts are sufficiently large (mean $=1181$, minimum $=681$) to use a log-Gaussian model as an approximation to the Poisson. We include covariates for (natural logarithm of) CDW population count, $P^{CDW}_{it}$, and measurable sources of error namely time and proportion of LSOA population registered at a GP not included in the CDW data sharing agreement (calculated using NHS Digital data). A generalised linear model (GLM) was first explored. The residuals exhibited strong spatio-temporal correlation: Moran’s I statistics computed on the residuals for each year separately produced values ranged from 0.23 to 0.33 with *p*-values less than 0.0001 in all years while the lag-1 temporal autocorrelation calculated for each LSOA separately yielded a mean of 0.3762 across all LSOAs.

We consider a model that captures the spatio-temporal autocorrelation via random effects assigned a spatio-temporal extension of conditional autoregressive (CAR) distributions, which are a type of Gaussian Markov random field. We assume the random effects to represent the unmeasured error in the CDW counts. Here we follow the model proposed by Rushworth et al. [40]. Let $S = \left( S_{1},\ldots ,S_{T+1}\right)$ denote the set of random effects for time points $t=1,\ldots ,T+1$, where $S_{t} = \left( S_{1t},\ldots ,S_{Nt}\right)$ is the vector of random effects for specific time point *t*. Then,

$$\begin{aligned} \text {log}(P^{NHS}_{it}){} & {} \sim \text {N}\left( x_{it}^{\top }\beta + S_{it}, \sigma ^{2} \right) \\ S_{t}|S_{t+1}{} & {} \sim \text {N}\left( \rho _{T} S_{t+1} , \tau ^{2} Q(\rho _{S}, W)^{-1} \right) \qquad \; (t=1,\ldots , T)\, \\ S_{T+1}{} & {} \sim \text {N}\left( 0, \tau ^{2} Q(\rho _{S}, W)^{-1} \right) \, . \end{aligned}$$

The vector $x_{it}$ denotes the set of explanatory variables, $\beta$ the corresponding regression parameters, and $\sigma ^{2}$ the variance of the residual errors. For the distributions of the random effects, $\rho _{T}$ denotes the temporal dependency parameter, $\rho _{S}$ the spatial dependency parameter, $\tau ^{2}$ the conditional variance parameter, *W* an $N \times N$ neighbourhood matrix defined for the 204 non-overlapping spatial units that comprise the lattice data for this study, and *Q* the Leroux precision matrix [41]. Further detail for the spatio-temporal CAR model methodology can be found in the Supplementary material.

The random effect for time point $T+1$ is specified marginally since $S_{T+2}$ is not observed. A typical first-order autoregressive process defines each value conditioned on the previous value. We condition in the reverse order since data is extracted from the CDW retrospectively making the most recent data the most accurate and error accumulating as we go further back in time.

#### Modelling referrals to outpatient respiratory clinics

Let $Y_{it}$ be the number of new referrals from GP to an outpatient respiratory clinic for LSOA *i* ($i=1,\ldots ,N$) and year *t* ($t=1,\ldots ,T$). The referral data is modelled using a Poisson generalised linear mixed model (GLMM) with a random intercept term for each LSOA, denoted by $Z_{i}$. The adjusted number of CRD patients from the first part of the methodology, $\hat{R}_{it}$, is included as an offset term to give a rate interpretation. Then,

$$\begin{aligned} Y_{it}{} & {} \sim \text {Poisson}\left( \hat{R}_{it}\exp \left( d_{it}^{\top }\gamma + Z_{i}\right) \right) \\ Z_{i}{} & {} \sim \text {N}\left( 0, \kappa ^{2}\right) \, , \end{aligned}$$

where $d_{it}$ is the vector of explanatory variables, $\gamma$ the corresponding regression parameters, and $\kappa ^{2}$ the variance of the random effects for which we assume independence. A corresponding Poisson GLM was over-dispersed yet exploratory analysis carried out on the residuals did not provide evidence to support a more complex correlation structure for the random effects (details given in the Supplementary material file).

The covariate component of the GLMM is:

$$\begin{aligned} d_{it}^{\top }{} & {} \gamma = \gamma _{0} + \gamma _{1}\,\texttt {Age}^{65-74}_{it} + \gamma _{2}\,\texttt {Age}^{75+}_{it} + \gamma _{3}\,\texttt {Male}_{it} + \gamma _{4}\,\texttt {Distance}_{i} \\{} & {} + \gamma _{5}\, \texttt {IMD}_{i} + \gamma _{6-12}\,\texttt {Year}_{t} + \gamma _{13}\,\texttt {MBRN}_{it} + \gamma _{14-20}\,\texttt {Year}_{t}*\texttt {MBRN}_{it} \; . \end{aligned}$$

The covariates Age^65-74^, Age^75+^, and Male are included to account for demographic differences in the LSOAs and respectively represent the proportion of the adult population in the 65-74 and $75+$ age brackets, and proportion of the adult population that are male. Stepwise covariate selection (with age groups 25-39, 40-54, 55-64, 65-74, 75+) suggested the age groups included are the only ones that are predictive of referrals and have a distinct effect to each other. Distance represents the average car travel distance to the nearest hospital within the MBCCG providing respiratory outpatient services. IMD represents the Index of Multiple Deprivation (IMD) scores where a higher score indicates greater levels of deprivation. The IMD is updated every 3-4 years thus we take the mean of the 2015 and 2019 scores for each LSOA.

To account for the effect of MBRN intervention, we calculate the percentage of an LSOAs GP-registered population that is registered at a GP that joined the MBRN in 2017, represented in the model by MBRN. This is calculated for all study years , even prior to MBRN introduction, to account for any baseline differences in health service utilisation for LSOAs that received MBRN intervention from 2017 onward. For the purpose of exploratory data analysis, we dichotomise the continuous MBRN variable so that an LSOA is classed as an “MBRN LSOA” if MBRN $>50\%$ and a “Non-MBRN LSOA” otherwise.

Year represents the study year and MBRN*Year is an interaction term between study year and MBRN coverage, which will be the main indicator of the impact of the MBRN on outpatient referrals. Study year has been defined as a factor variable as opposed to a continuous covariate or a before/after MBRN indicator, in order to better study the evolution of MBRN impact since its initiation. For the sake of space, the factor levels have been grouped into one term in the above equation.

Additional descriptions of covariates used for both models can be found in the Supplementary material file.

#### Inference

The models are specified as Bayesian hierarchical models and parameter estimation carried out using Markov Chain Monte Carlo (MCMC) algorithms. For the spatio-temporal GP registration model, prediction for years 2012 and 2013 is carried out as part of model fit. The unobserved data are treated as missing values in the response vector and are estimated each iteration of the MCMC algorithm via the posterior predictive distribution to produce a posterior sample. When fitting the referrals model, to account for the uncertainty in the predictions, we randomly sample from the posterior samples for the predictions each iteration and recalculate the offset term. For further information on MCMC specifics, including prior distributions, we refer readers to the Supplementary material file. The significance of model covariates is tested at the 5% significance level using Bayesian credible intervals (CIs). A covariate is insignificant if the interval contains the null value. All statistical analysis was carried out in R Studio [42].

## Results

### Adjusting CRD patient count

**Fig. 2:**
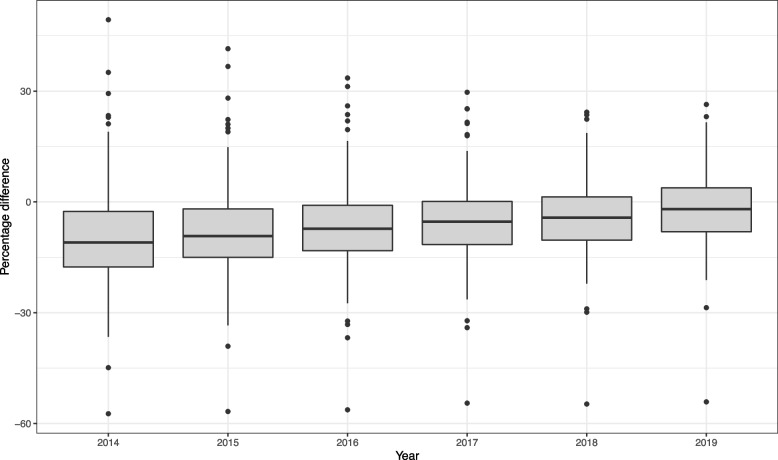
Boxplot showing the spread of relative difference between the CDW counts and NHS Digital estimates for adults registered at a MBCCG GP at LSOA-level. Percentage difference $= (\text {CDW} - \text {NHS})/\text {NHS} \times 100$. Data for years 2012 and 2013 not available from NHS Digital

Since the spatio-temporal model for patient count adjustment is not the main focus of this paper, we refer the interested reader to the Supplementary material for extended results including covariate description, parameter estimates, prediction output, model validation, and MCMC diagnostics. Figure 2 and Table 1 are included here to highlight respectively the need and impact of the proposed adjustment modelling.

Figure 2 shows the spread of percentage change between the CDW GP-registered population counts and NHS Digital estimates for study years 2014-2019. As we go further back in time, the magnitude of the median percentage difference increases and there is increased variation in the degree of error. The plot shows an LSOA that is consistently a 50-60% underestimate in the CDW whilst other LSOAs have above a 30% overestimate in years 2014-2016, highlighting the error that can occur at both ends of the spectrum using CDW registration data.

Table 1 summarises the overall impact of the adjustment methodology on the total number of CRD patient counts that is used as the denominator of the outpatient referral rate.

**Table 1:** Comparison of unadjusted (raw counts extracted from CDW) and adjusted (prevalence $\times$ GP-registered adult population) total CRD patients within the MBCCG for each study year

Year	Unadjusted	Adjusted	Percentage difference
2012	22,803	26,293	$+15.3\%$
2013	23,747	26,962	$+13.5\%$
2014	24,876	27,820	$+11.8\%$
2015	25,936	28,477	$+9.8\%$
2016	27,082	29,305	$+8.2\%$
2017	28,234	29,975	$+6.2\%$
2018	29,262	30,631	$+4.5\%$
2019	30,902	31,715	$+2.6\%$

### Modelling referrals to outpatient respiratory clinics

#### Raw data

We first present data summary results. Table 2 shows a comparison of age, sex, distance to nearest hospital, and IMD score for LSOAs that received MBRN intervention in 2017 and those that did not. Populations of the MBRN LSOAs are on average younger, closer in distance to a major hospital, and have higher relative deprivation.

**Table 2:** Descriptive statistics of covariates used in the outpatient referrals random intercept model for MBRN and non-MBRN intervention LSOAs

	MBRN *Median (interquartile range)*	Non-MBRN *Median (interquartile range)*	Difference
Age 65-74	15.4 (12.1, 19.3)	18.5 (14.7, 21.0)	-3.1
Age 75+	12.6 (9.1, 16.6)	14.0 (11.3, 17.0)	-1.4
Male	47.7 (46.5, 49.0)	48.1 (46.8, 49.6)	-0.4
Distance	6.0 (2.4, 8.2)	11.0 (3.76, 20.4)	-5
IMD	19.5 (11.8, 31.6)	14.7 (10.0, 22.5)	4.8

A total of 8,897 referrals to outpatient respiratory clinics that fulfilled the inclusion criteria were extracted from secondary care records in the CDW. Table 3 documents the raw counts by study year and the average number of referrals per LSOA. The total number of new referrals from GP to respiratory outpatient clinics displayed a consistent increasing trend up to 2016, but the counts in the years since the introduction of the MBRN (2017-2019) have not risen above 2016 levels.

**Table 3:** New referrals from GP to outpatient respiratory clinics for each study year

Year	Total number of referrals	Average per LSOA
2012	968	4.75
2013	974	4.77
2014	1,039	5.09
2015	1,120	5.49
2016	1,218	5.97
2017	1,204	5.90
2018	1,165	5.71
2019	1,209	5.93

Additional data summaries can be found in the Supplementary material.

#### Model output

Table 4 presents the parameter estimates for the GLMM.

The main indication of the effect of the MBRN are the interaction terms between MBRN coverage and year. Prior to MBRN intervention (2012-2016), the model output does not suggest a systematic difference in referral rates at baseline, after adjusting for all other covariates, for LSOAs that received higher percentages of MBRN intervention from 2017 onward. The MBRN main effect term (i.e., the effect in 2012) and the interaction term for 2015 are marginally significant, whilst the interactions terms for 2013, 2014, and 2016 are insignificant, at the 5% significance level.

The MBRN did not have a significant association with referral rate in the activation year (2017). In 2018, a 1% increase in percentage of the population registered at an MBRN GP was associated with a 0.04% decrease in rate of referral to outpatient respiratory clinics from GP. To put this figure in context, an LSOA with all its population registered at an MBRN GP (i.e., full intervention, $\texttt {MBRN}=100\%$) is associated with a 31.8% (95% CI 17.0-43.9) decrease in referral rate compared to an LSOA with none of its population registered at an MBRN GP (i.e., no intervention, $\texttt {MBRN}=0\%$), with all other covariates held constant. In 2019, the same 1% increase is associated with a 0.05% decrease in referral rate, corresponding to a 40.5% (95% CI 27.5-50.9) decrease in referral rate for full MBRN intervention compared to no intervention.

The model output does not suggest a significant change in overall referral rate over time beyond what can be attributed to changes in demographic factors and the introduction of the MBRN. All levels of the year factor variable are insignificant except for 2016 which shows a marginally significant 9.1% increase in referral rate compared to 2012.

**Table 4:** Median fitted covariate values for the outpatient referrals random intercept model

Parameter	RR	95% CI
Intercept	0.037	(0.035, 0.040)
Age 65-74	1.017	(1.008, 1.026)
Age 75$+$	1.009	(1.001, 1.016)
Male	1.016	(1.002, 1.028)
Distance to hospital (km)	1.005	(1.001, 1.009)
IMD	0.998	(0.996, 0.999)
2013	0.965	(0.878, 1.054)
2014	0.991	(0.909, 1.078)
2015	1.030	(0.950, 1.128)
2016	1.091	(1.000, 1.191)
2017	1.049	(0.963, 1.149)
2018	0.975	(0.894, 1.064)
2019	0.961	(0.880, 1.049)
MBRN (*main effect*)	1.001	(1.000, 1.003)
MBRN 2013	0.998	(0.996, 1.000)
MBRN 2014	0.999	(0.997, 1.001)
MBRN 2015	0.998	(0.996, 1.000)
MBRN 2016	0.999	(0.997, 1.001)
MBRN 2017	0.999	(0.997, 1.001)
MBRN 2018	0.996	(0.994, 0.998)
MBRN 2019	0.995	(0.993, 0.997)

*RR* relative risk, *CI* credible interval

**Fig. 3:**
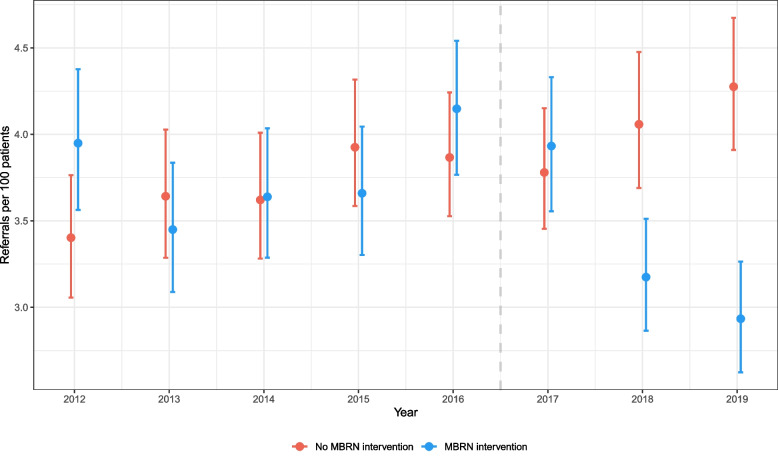
Interaction plot for the effect of the interaction term between year and MBRN on referral rate over time. The predicted number of referrals per 100 CRD patients is estimated for two levels of registration at an MBRN GP at LSOA-level: 0% (“No MBRN intervention”) and 100% (“MBRN intervention”). The dashed line represents the introduction of the MBRN in 2017

Figure 3 is an interaction plot providing an illustration of the effect of MBRN intervention over time. The plot is produced using the fitted model output and compares the predicted referral rate for an LSOA with full MBRN intervention (MBRN=100%) compared to an LSOA with no MBRN intervention (MBRN=0%). The rate of referral is predicted for each study year whilst all other covariates including the offset term are fixed at their median values across the entire data set. In the baseline years (2012-2016) and in the activation year (2017), the credible intervals for the predictions consistently overlap, illustrating no systematic difference between intervention and non-intervention areas once all other covariates adjusted for. In 2018 and 2019, the intervals separate, with the non-intervention LSOA continuing in an upward trend and the intervention LSOA substantially decreasing. In 2019, the model predicts a median rate of 2.9 referrals per 100 CRD patients (95% CI 2.6-3.3) for full MBRN intervention compared to 4.3 per 100 CRD patients (95% CI 3.9-4.7) for no MBRN intervention.

All other covariates included in the model are significant (Table 4). The covariates relating to age and sex demographics are positively associated with referral rate. For each 1% increase in the proportion of an LSOAs adult population in the 65-74 age bracket, a 1.7% increase in referral rate would be expected. For the 75+ age bracket, the analogous increase in referral rate is 0.9%. For each 1% increase in the proportion male, a 1.6% increase in referral rate would be expected. Distance to closest hospital is also positively associated with referral rate with an increase of 0.5% associated with a 1 km increase in road travel distance. The model results suggest a negative relationship between referral rate and socio-economic deprivation. A 1 point increase in IMD score is associated with a 0.02% decrease in referral rate, equating to a referral rate 1.2 times higher for the least deprived LSOA in the study area compared to the most deprived.

The variance term for the random effects, $\kappa ^2$, was estimated at 0.023, 95% CI (0.015, 0.033), supporting the need for a GLMM over a GLM.

Diagnostics for the MCMC algorithm can be found in the Supplementary material.

## Discussion

Integrated care is a broad concept with multi-faceted implications which presents a challenge for evaluators. This study considered the use of routinely collected data to provide a robust data-driven analysis of healthcare delivery that focuses on an outcome measure of relevance and adjusts for diversity in the study population. The results suggest the success of the MBRN model in reducing rates of new referrals to outpatient respiratory clinics from GP in areas that have received higher percentages of intervention compared to areas with lower intervention. Three years of full MBRN intervention was associated with a 40.5% decrease in referral rate, adjusted for changes in CRD patient count [14, 43].

The first stage of the methodology in this paper applies existing saptio-temporal methodology to a new setting to model official statistics and predict beyond the published time frame at the required geography level based on error-filled routine data. In addition, we account for uncertainty in the predictions by using the full posterior predictive distributions in the model fit for the mixed Poisson model. The results from the first model illustrate the consequences of the issues described with the CDW GP registers. For example, LSOAs where a large proportion of the population is registered at a GP not in the CDW data sharing agreement can result in substantial underestimates of the true GP-registered population. In contrast, LSOAs that have undergone significant housing development can have an overestimate of the true GP-registered population in years prior to the building work. If individuals move into the houses from the local area, the CDW does not store the address history, thus assigning them to an address at a time before the housing existed. The methodology we have proposed could be used in other fields of research that use time restricted official statistics such as further areas of health service provision, public health and social care, and the broader social sciences.

This research into outpatient referrals supports findings from systematic reviews that integrated care services for a specific chronic disease using an MDT approach with disease-specific specialist input is likely to be successful at reducing hospital activity [11, 44]. However, existing evaluations of integrated services for chronic respiratory disease commonly focus on COPD alone, and often target only the most high-risk patients to prevent non-elective hospital admissions [26–28]. The MBRN adds to the existing literature by showing the potential for effective integrated care with a broader patient scope and benefits to other aspects of healthcare service utilisation, namely outpatient attendances. It is not a given that empowering primary care will decrease outpatient service usage since integrated care initiatives can identify unmet needs in their populations, resulting in evaluations reporting an increase in total healthcare service usage [30]. In addition, an often cited limitation of integrated care evaluations is the short follow-up period [11], an issue found in the literature that considered the impact of enhanced primary care models on referrals. In this analysis, the effect of the MBRN progressively increased between 2017-2019 highlighting the importance of evaluating healthcare initiatives over sufficient time periods [14, 43]. Policymakers frequently want to see immediate results yet transformational changes in practice and work culture requires time to gain traction [45].

The model results identified a negative relationship between socio-economic deprivation and rate of referrals, after adjustment for CRD patient count. This finding supports existing literature that the most disadvantaged patient groups often have lower probabilities of attending specialist care [46, 47]. This study is unable to comment on the reason for the inequality in the MBCCG context; possible reasons include patient preference [46], lack of adequate communication or health literacy [48, 49], or differences in GP referral behaviour across the study area [50]. Existing research predominately does not find an association between socio-economic position and probability of visiting primary-care in developed countries, with some studies even reporting higher rates of attendance [46, 51]. There is an opportunity for integrated care services to reduce healthcare inequalities by training primary care to provide more specialist services.

Of the remaining covariates in the model, the 65-74 age bracket was at the greatest risk of higher rates of referrals followed by proportion male. Distance to hospital was positively associated with rate of referral, which contrasts with existing research that links rurality with reduced access to services [52]. A probable explanation for this finding is the relative affluence of the rural areas in the MBCCG. We are unable to identify previous work in which outpatient referrals have been analysed at an LSOA-level in England. The study area contained 32 GPs but 204 LSOAs, hence using the small-area geography gave potential for insight into the contribution of other risk factors for rate of referral that may have been lost if covariates were averaged to GP-level. This is a particular issue in the MBCCG where several practises are made up of multiple sites. For example, Lancaster Medical Practice is comprised of eight separate sites spread over central Lancaster, serving a wide spectrum of patients, demographically and clinically speaking.

The recovery of elective care following the COVID-19 pandemic is not unique to the NHS but is affecting healthcare systems worldwide [53]. The NHS post-COVID recovery plan states the need for an increase in activity of 30% above pre-pandemic level by 2024/25 to reduce waiting times, but this goal has been met with scepticism in light of NHS staff shortages and recruitment challenges [24, 54, 55]. The MBRN model demonstrates that effective integrated care has real potential to optimise existing services and ease the burden on respiratory outpatient services by reducing the need for an onward referral through improved patient pathways, effective communication between healthcare tiers, and an upskilled primary care team. The results of this analysis suggest a potential reduction of 1.4 referrals per 100 CRD patients per year for an LSOA with full MBRN intervention comapred to no MBRN intervention. Applying this result to the MBCCG population that had an estimated 31,715 adults with a CRD diagnosis in 2019 (Table 1), this would equate to a difference of over 400 referrals a year; 930 referrals under full MBRN intervention compared to 1,356 under no MBRN intervention. Assuming a respiratory clinician has 2-3 4-hour clinics per week and assigns 30 minutes to a new patient [56], the reduction of over 400 new referrals per year in the MBCCG population would equate to approximately one fewer clinics per week, with no consideration made for the knock-on effect to follow-up appointments.

A key strength of the proposed methods is using number of diagnosed CRD patients as the referral rate denominator. Disease prevalence data is not always readily available particularly at small area geography level and changes in patient counts, beyond what is able to be accounted for through population growth and known risk factors, can distort both space and time analyses of healthcare utilisation [57, 58]. The model in this paper controls for changes in the size of the patient cohort, allowing a closer study of the underlying referral behaviour.

Data is vital for understanding the impacts of health interventions and generating robust analytics to improve healthcare delivery [43, 59]. Access to the CDW facilitated the flexibility of this analysis and key strengths of the methodology, including choice of outcome measure, spatial unit, adjusting for changes in CRD patient size, a longer study period, and filtering referrals and patients at a finer scale to capture healthcare interactions of closest relevance to the MBRN. This research is the first extensive use of the CDW for health service research and has barely scratched the surface of its potential. The CDW uses pseudonymised NHS Numbers; linking data at a patient level and removing traditional data silos between different branches of healthcare has the potential to provide a far more realistic and holistic view of patient care pathways. There is a clear, high value in investing in databases and personnel to exploit the wealth of information available in routinely collected data to support evidence-based decision making [60, 61].

The limitations associated with routinely collected data are well-established [62, 63]. This research contributes to the existing literature by exploring limitations encountered when using primary care records for space-time analyses, particularly the difficulty in tracking movement of people. The methodology proposed to circumvent the issues identified is somewhat of a crude fix and relies on the assumption that the prevalence calculated from the CDW is representative of the true, unobserved adult population for the corresponding space-time unit. This assumption may not be reasonable for error introduced by movement of people due to the relationship between transiency and age. If an age breakdown was provided at LSOA-level by NHS Digital, then a more informed adjustment to CRD patient count could be considered. Nevertheless, the strong spatio-temporal correlation identified in the CDW error process may be useful for future research into methodology for improving analysis using routinely collected healthcare data.

Other limitations of this research must be recognised. First, this is not a controlled study. The data summary results evidence that the MBRN reaches the most urbanised and deprived areas of the MBCCG. Access to a larger national or sub-national routine data source would facilitate a matched controlled study. However, exploratory analysis (found in the Supplementary material) and the interaction terms in the GLMM prior to MBRN introduction did not suggest a systematic difference in referral rates, once all else adjusted for, between intervention and non-intervention areas. Therefore, it is reasonable to attribute the dramatic decrease in referrals in 2018 and 2019 to the work of the MBRN. However, areas that have not received MBRN intervention may not have the same capacity for referral reduction due to potential differences in disease severity and patient need, which are not accounted for in the model. Second, in the GLMM, year is defined as a factor variable, adding to model complexity and forcing the relative risks to be compared to a baseline year, in this case 2012. The factor variable was selected as it captures the evolving and distinct impact of the MBRN in each intervention year. In contrast, other representations of time, such as a linear time trend or a before/after indicator variable, would assume a fixed trend across larger time periods. Due to the small temporal sample size, more advance time series modelling was not appropriate. Third, the study period was restricted by the introduction of the first COVID-19 lockdown in England, so we cannot comment on whether the reduction in referral rate was sustained. Finally, reason for referral is not documented in the secondary care records in the CDW therefore it is likely that the referrals modelled in this paper include irrelevant referrals. Access to unstructured data, such as referral letters, may minimise this source of bias. However, the clinic inclusion-exclusion criteria were determined in consultation with MBRN physicians to best capture referrals that aligned with MBRN prioritie.

Future research should explore other impacts of the MBRN integrated care initiative. This analysis has focused on outpatient referrals given the relevance to MBRN goals and current healthcare pressures, as well as the gap in the integrated care evaluation literature. However, it is important to consider other measures, such as patient experience, standard of care received in primary care, and health outcomes, to provide a full-picture evaluation of an integrated care initiative. The associated cost to an initiative is a critical factor in decision-making for policy makers. The cost savings associated with reduced outpatient referrals have not been included in this study as these results may be misleading. The MBRN model has many facets beyond efficient referral pathways, thus a separate cost-effectiveness analysis that captures the initiatives complexity would be required.

### Conclusion

Overall, our novel analysis demonstrates the use of large routinely collected data to robustly evaluate key outcome measures of integrated care, in this case, rate of referrals to outpatient services. The results of this study are of great relevance to current healthcare pressures across the globe, with large outpatient service backlogs and demand for innovative models of care. Future work should focus on assessing other measures appropriate to the MBRN to provide a full evaluation of the initiative’s model of care.

### Supplementary Information

**Supplementary material 1.**

## Data Availability

The primary data of this study is routinely collected NHS data provided by the University Hospitals Morecambe Bay NHS Foundation Trust. The data is not publicly available due to patient data confidentiality and restrictions imposed by the Health Research Authority (HRA). Permission to access this data must be granted by the HRA. However, other socio-economic and demographic data are publicly available. Website links for raw data extract can be found in the references. The aggregated forms used for this analysis can be made available from the corresponding author upon reasonable request.

## Abbreviations

CAR: conditional auto-regressive
CDW: Community Data Warehouse
COPD: chronic obstructive pulmonary disease
CRD: chronic respiratory disease
GLLM: generalised linear mixed model
GLM: generalised linear model
GP: general practice
ILD: interstitial lung disease
IMD: Index of Multiple Deprivation
Km: kilometres
LSOA: Lower-layer Super Output Area
MBCCG: Morecambe Bay Clinical Commissioning Group
MCMC: Markov chain Monte Carlo
MBRN: Morecambe Bay Respiratory Network
NHS: National Health Service
ONS: Office for National Statistics
