# Supplementary material for: Spatio-temporal modelling of referrals to outpatient respiratory clinics in the integrated care system of the Morecambe Bay area, England
Source: BMC Health Serv Res. 2024 Feb 22;24:229. doi: 10.1186/s12913-024-10716-7 (PMC10882730; doi:10.1186/s12913-024-10716-7)
Supplement: Supplementary file 1 — Supplementary material 1. [file 12913_2024_10716_MOESM1_ESM.pdf]

Supplementary material for: Spatio-temporal modelling of referrals to outpatient respiratory clinics in the integrated care system of the Morecambe Bay area, England

Rachael Mountain, Jo Knight, Kelly Heys, Emanuele Giorgi, and Timothy Gatheral

Contents

1 Primary data source **2**

2 GP registration model **3**

2.1 Model motivation 3

2.2 Exploratory analysis 3

2.3 Methodology 4

2.3.1 Model specification 4

2.3.2 MCMC methodology 7

2.4 Results 9

2.4.1 Model output 9

2.4.2 MCMC diagnostics 11

3 Outpatient referrals model **15**

3.1 Exploratory data analysis 15

3.2 MCMC methodology 20

3.3 MCMC Diagnostics 22

Section 1 provides further detail of the steps that were taken when constructing the analysis data set from the Community Data Warehouse (CDW). Section 2 contains additional information of the spatio-temporal general practice (GP) registration model that was omitted from the main article as this is not the primary focus of the research. Finally, Section 3 contains exploratory data analysis, Markov Chain Monte Carlo (MCMC) methodology, and diagnosis, to supplement the information given in the main article.

1 Primary data source

The construction of the population data set from the CDW is outlined in the Methods section of the main article. Here we provide additional detail of decisions made, for the sake of transparency:

- Duplicate NHS Numbers (most commonly caused by an individual being registered at more than one GP) with agreeing sex, date of birth, and date of death (if applicable), are assumed to be the same person, and the record with the most recent GP registration start date is taken as their current GP practice and address. If there is discrepancy then all records with the given NHS number are excluded.

- In order to be counted in a particular year, an individual's entry date must be prior to the half way point (1st October) of the given year and their end date after the halfway point. This was done to avoid overestimating the population count, particularly in areas with highly transient populations.

- For a given GP registration in the CDW, only the individual's current address, rather than entire address history, is recorded. A change in address can only be identified if it is accompanied by a change in registered GP. Consequently, it is possible to observe "large" moves in people, but not "smaller" local moves. This limitation is exacerbated by several GP practices in the Morecambe Bay Clinical Commissioning Group (MBCCG) being made up of multiple sites. For example, Lancaster Medical Practice is comprised of eight separate sites spread over central Lancaster, hence an individual could move multiple times living in varied areas, demographically speaking, whilst remaining with the same GP. In addition, since GP registration end date is missing, it is not possible to determine whether there are breaks between registrations, for example if an individual has moved out of the MBCCG then moved back in at a later date. Therefore, we only consider the most recent registration for each distinct individual. Although this does waste some information, given the relatively short length of the study period it should only impact majorly on areas with transient populations which will have a spatial correlation.

- "Regular" registrations only are considered. In England, a "temporary" GP registration can be used whilst away from home for work, study, or on holiday, for up to three months. Individuals with a temporary registration remain registered with their permanent GP surgery during this time.

2 GP registration model

The primary focus of the main article is referrals to outpatient respiratory clinics and the impact of the MBRN, hence minimal focus is given to the extensive work carried out to model the GP-registered population. In this section we provide extended detail of the model, including motivation, exploratory analysis, methodology, and results. Some information from the main article has been duplicated for the sake of clarity and completeness.

2.1 Model motivation

For our study into referrals to outpatient respiratory clinics, it is crucial to adjust for patient burden to avoid producing a model that simply acts as a proxy for chronic respiratory disease (CRD) prevalence. Our modelling approach includes CRD patient count as an offset term to express the rate of referral for each space-time unit in terms of number of CRD patients. Patients with a CRD diagnosis are identified from CDW primary care records. However, the number of patients registered at a MBCCG GP for a given space-time unit is open to multiple sources of error which will in turn bias the CRD patient count. NHS Digital has released GP-registered population data at Lower Layer Super Output Area (LSOA) level since 2014, allowing us to apply a correction to our CRD patient count for study years 2014-2019 (see Methods: Statistical Analysis: *1. Adjusting CRD patient count* of the main article). However, NHS Digital has only released this data since 2014, whereas our study begins in 2012. The purpose of this model is to model the error process between the true GP-registered population count taken from NHS Digital and the corresponding erroneous population count from the CDW. The model output allows us to predict the unobserved NHS Digital data for 2012 and 2013 and apply the correction to the CRD patient count. For our research into outpatient referrals, the GP registration model is a means to an end, but the methodology outlined could be applied to other research where official statistics are required beyond the time frame for which they have been reported.

2.2 Exploratory analysis

The outcome variable, denoted P_{it}^{NHS}, is the number of adults ≥ 25 years registered at an MBCCG GP for LSOA $i = 1, \ldots, N$ and year $t = 1, \ldots, T+1$, calculated from NHS Digital. Our study of outpatient referrals is over $T = 8$ study years (2012-2019), yet for this model we utilise data from the year 1st April 2020 - 31st March 2021. We recognise that including the 2020/21 year may introduce COVID-related bias (e.g., people may have been less likely to move house or relocate during the pandemic which would in turn affect GP registration) but it has been included to increase the sample size and improve the model's prediction capacity.

We first consider a generalised linear model (GLM). The outcome counts are sufficiently large (mean = 1181, minimum = 681) to justify a log-Gaussian model as an approximation to the Poisson. The natural logarithm of CDW population count, from which we aim to predict the outcome variable for the unobserved years, is included as a covariate ($\log(\texttt{CDW})$). Additional covariates are included to

adjust for known sources of error, namely year ($\texttt{Year}$) and proportion of the population registered at a GP not included in the CDW data sharing agreement ($\texttt{Missing}$). Table S1 provides further details of the variables used in analysis and Table S2 provides summary statistics. The GLM is of the form:

$$\log(P_{it}^{NHS}) = \beta_0 + \beta_1 \log(\texttt{CDW}_{it}) + \beta_2 \texttt{Year}_t + \beta_3 \texttt{Missing}_{it} \,.$$

Spatial autocorreltion was explored using Moran's I statistic computed on the GLM residuals for each year separately; the values ranged from 0.23 to 0.33 with p-values<0.0001 in all years. The lag-1 temporal autocorrelation calculated for each LSOA separately yielded a mean of 0.3762 across all LSOAs. Therefore, we pursued modelling approaches that would account for strong spatio-temporal correlation.

Table S2: Summmary statistics for covariates used in the GP registration model (NHS Digital GP-registered population, CDW population count, and proportion of the LSOA population registered at a GP not in the CDW).

	**Min**	**1st**	**Median**	**Mean**	**3rd**	**Max**
**NHS Digital**	680.6	960.5	1102.5	1180.7	1356.5	2210.7
**CDW**	365	881	1048	1095	1272	2153
**Proportion missing**	0	0	0	0.022	0.017	0.555

2.3 Methodology

2.3.1 Model specification

Let $S = (S_1, \dots, S_{T+1})$ be the set of random effects for time points $t = 1, \dots, T+1$, where $S_t = (S_{1t}, \dots, S_{Nt})$ is a vector of LSOA-level random effects for a given time point t. Then,

$$\log(P_{it}^{NHS}) \,|\, S_{it} \sim \mathrm{N}\left(x_{it}^T \beta + S_{it}, \sigma^2\right) \,,$$

where x_{it} is the vector of explanatory variables, β the corresponding regression parameters, and σ^2 the variance of the residual errors. The model captures the spatio-temporal autocorrelation by assigning the random effects a spatio-temporal extension of conditional autoregressive (CAR) priors, which are a type of Gaussian Markov random field (GMRF). Here we follow the model proposed by Rushworth et al. [1],

$$\begin{aligned} S_t | S_{t+1} &\sim \mathrm{N}\left(\rho_T S_{t+1}, \tau^2 Q(\rho_S, W)^{-1}\right) \qquad t = 1, \dots, T \\ S_{T+1} &\sim \mathrm{N}\left(0, \tau^2 Q(\rho_S, W)^{-1}\right) \,. \end{aligned} \tag{1}$$

The S_{T+1} is specified marginally since S_{T+2} does not exist. We condition in the reverse order since the unobserved data is the furthest back in time as opposed to a future event which is more typically seen in prediction modelling.

The random effects specified in (1) are non-separable in space and time. Temporal correlation is modelled in the conditional expectation via a first-order autoregressive process with dependency

Table S1: Description of variables used in spatio-temporal GP registration model.

Variable	Time varying (Y/N)	Source	Description	Notes
GP-registered population	Y	NHS Digital	Average number of adults (25+ years) registered at a MBCCG GP for each LSOA inside the CCG boundaries	LSOA-level data released quarterly (1st Jan, 1st Apr, 1st Jul, 1st Oct) since January 2014. LSOA-level given for all-age only. GP-level given in five-year age brackets. Number 25 years or over estimated for each LSOA by multiplying the number registered at each GP by the proportion of that GPs register over 25. Estimates calculated for each quarter and averaged across study years.<br>Only consider data for MBCCG GPs and LSOAs within the MBCCG.<br>'Regular' registrations only. 'Temporary' registrations are not counted in the NHS Digital data
CDW GP register counts	Y	CDW (primary care records)	Annualised count of the number of adults (25+ years) registered at a MBCCG GP for each LSOA inside the CCG boundaries	Entry date – most recent of study start date (01/04/12), 25th birthday, and GP registration start date.<br>End date – earliest of date of death and GP registration end date proxy. If not relevant then 'NA'.<br>'Regular' registrations only. 'Temporay' registrations not included.
Year	Y	NA	Continuous variable form of year	Exploratory analysis suggested a linear trend between (natural logarithm of) GP-registered population and time, hence the use of a continuous form of year.

Missing GPs	Y	NHS Digital	Percentage of the LSOAs GP-registered population missing from the CDW as a result of GPs not in the data sharing agreement	Two GPs not in the data sharing agreement of the CDW. An additional GP closed in September 2015 (before the CDW was created), patients had to register at a new GP so these patients are "missing" pre-September 2015.<br>Percentage calculated using LSOA-level GP registration data released by NHS Digital. We do with calculations with all-age data and assume this variable not to be correlated with age. As with the 'GP-registered population' variable, mean taken across quarters.<br>For the study years 2012 and 2013, the 2014 value is used. Exploratory analysis suggests this variable does not fluctuate year-on-year.

parameter ρ_T, whereas the spatial autocorrelation is induced via the precision matrix, Q. Numerous specifications for the precision matrix have been proposed in the CAR literature, but here we use that proposed by Leroux et al. [2], $Q(\rho_S, W) = \rho_S W + (1-\rho_S)I$ where ρ_S is the spatial dependency parameter, I the $N \times N$ identity matrix, and W an $N \times N$ neighbourhood matrix defined for the 204 non-overlapping spatial units that comprise the lattice data for this study. Using the notation $i \sim j$ to mean "areas i and j share a common border" and n_i to be the total number of neighbours for area i, the individual elements of W are:

$$w_{ij} = \begin{cases} n_i & \text{if } i = j \\ -1 & \text{if } i \sim j \\ 0 & \text{otherwise}\,. \end{cases}$$

Thus the precision matrix is a weighted average of the spatially dependent and independent structures, accommodating both weak and strong spatial autocorrelation [3]. The univariate full conditional distribution better illustrates the spatial relationship, and is given by,

$$S_{it}|S_{-i,t} \sim \mathrm{N}\left(\frac{\rho_S \sum_{j\sim i} S_{jt}}{n_i\rho_S + 1 - \rho_S}, \frac{\tau^2}{n_i\rho_S + 1 - \rho_S}\right)\,.$$

The subscript notation $_{-k}$ is used to mean "all elements except k", and so $S_{-i,t}$ is the vector of all random effects at time point t excluding area i.

2.3.2 MCMC methodology

Model fit was carried out by sampling from the posterior distribution of the parameters using MCMC methodology. It is assumed the reader has an understanding of the fundamentals of Bayesian inference and MCMC methods.

Prior distributions:

The random effects, S_{it} ($i = 1, \ldots, N$ and $t = 1, \ldots, T+1$), act as latent variables and the spatio-temporal CAR prior is described in (1). For the remaining parameters, we assume the following independent prior distributions:

$$\begin{aligned} \tau^2 &\sim \text{Inverse-Gamma}\,(a, b) \\ \sigma^2 &\sim \text{Inverse-Gamma}\,(c, d) \\ \rho_T &\sim \text{Unif}\,(0, 1) \\ \rho_S &\sim \text{Unif}\,(0, 1) \\ \beta &\sim \mathrm{N}\left(0, \lambda^2 I_4\right)\,, \end{aligned}$$

where $a = c = 1$, $b = d = 0.01$, $\lambda^2 = 1000$, and I_4 the 4-dimensional identity matrix.

Posterior distribution:

Let $\theta = (\beta, S, \tau^2, \sigma^2, \rho_T, \rho_S)$ be the vector of parameters to be estimated. The joint posterior distribution is:

$$
\begin{aligned}
\pi\left(\theta|P\right) &\propto f\left(\log\left(P\right)|S\right) \times \prod_{t=1}^{T+1} \pi(S_t|S_{t+1}) \times \pi(\tau^2, \sigma^2, \rho_T, \rho_S, \beta) \\
&\propto \left(\sigma^2\right)^{-N(T+1)} \exp\left(-\frac{1}{2\sigma^2}\left(\log(P) - X\beta - S\right)^T \left(\log(P) - X\beta - S\right)\right) \\
&\quad \times det\left(\tau^2 Q^{-1}\right)^{-(T+1)/2} \exp\left(-\frac{1}{2\tau^2}\sum_{t=1}^{T+1}\left(S_t - \rho_T S_{t+1}\right)^T Q \left(S_t - \rho_T S_{t+1}\right)\right) \\
&\quad \times \pi(\tau^2, \sigma^2, \rho_T, \rho_S, \beta) \ .
\end{aligned}
$$

In the above we define $S_{T+2} = 0$ to simplify the posterior equation and write $\pi(\tau^2, \sigma^2, \rho_T, \rho_S, \beta)$ as shorthand for the prior distributions.

Updating algorithms:

The parameters $(\tau^2, \sigma^2, \rho_T)$ were updated via separate Gibbs samplers according to their full conditional posterior distributions:

$$
\begin{aligned}
\tau^2|P, \theta_{-\tau^2} &\sim \text{Inverse-Gamma}\left(a + \frac{N(T+1)}{2},\, b + \frac{1}{2}\sum_{t=1}^{T+1}\left(S_t - \rho_T S_{t+1}\right)^T Q \left(S_t - \rho_T S_{t+1}\right)\right) \\
\sigma^2|P, \theta_{-\sigma^2} &\sim \text{Inverse-Gamma}\left(c + \frac{N(T+1)}{2},\, d + \frac{1}{2}\left(\log(P) - X\beta - S\right)^T \left(\log(P) - X\beta - S\right)\right) \\
\rho_T|P, \theta_{-\rho_T} &\sim \text{Truncated-Normal}\left(\frac{\sum_{t=1}^{T+1} {S_t}^T Q S_{t+1}}{\sum_{t=1}^{T+1} {S_{t+1}}^T Q S_{t+1}},\, \frac{\tau^2}{\sum_{t=1}^{T+1} {S_{t+1}}^T Q S_{t+1}}\ ;\ 0, 1\right) \ ,
\end{aligned}
$$

where the parameters of the truncated normal distribution respectively correspond to the mean, variance, minimum value, and maximum value.

The parameter ρ_S was updated via a random walk Metropolis step. The tuning parameter was tuned to achieve an acceptance rate between 0.4 and 0.45.

The regression parameters, β, and latent variables, S, were updated jointly using Gaussian Markov random field (GMRF) full conditional sampling techniques outlined in Chapter 2 of Rue and Held (2005) [4]. To summarise, we define $\phi = (\beta, S)$ and so,

$$
\boldsymbol{\phi}|P, \theta_{-\boldsymbol{\phi}} \sim \mathrm{N}\left(\frac{1}{\sigma^2} V^{-1} A^T \log\left(P\right),\, V^{-1}\right) \ ,
$$

where A is the design matrix and V the precision matrix for ϕ. We omit the full specification of V^{-1}. To ensure V is singular, we enforce the linear constraint $\sum_i \phi_i = 0$. Sparse matrix methods combined with the algorithm for sampling from GMRFs under a linear constraint outlined on page 38 of Rue and Held (2005) were used to improve computational efficiency [4].

Inference

Inference was based on 2,000 independent samples obtained from 250,000 iterations of the algorithm with a burn-in of 50,000 and the remaining 200,000 thinned by a factor of 100 to remove any remaining autocorrelation. Convergence of the MCMC algorithm was established by the Gelman-Rubin convergence diagnostic calculated on three shorter chains to select a suitable length for the burn-in period. Trace plots, density curves, auto-correlation plots, and effective sample size (ESS) calculations were used to assess sufficient mixing of the final chain. The independent variables were standardised prior to model fit to reduce multicollinearity.

The unobserved data for years 2012 and 2013 (corresponding to years $t = 1, 2$) are treated as missing values in the response vector and are estimated each iteration of the MCMC algorithm according to the posterior predictive distribution:

$$\widehat{\log(P_{it})}|P \sim N\left(x_{it}^T\beta_0 + S_{it0}, {\sigma^2}_0\right) \qquad t = 1, 2 \ ,$$

where $_0$ has been used to denote the current values of the parameters at a given iteration.

2.4 Results

In preliminary runs of the MCMC algorithm, the temporal dependency parameter, ρ_T, was close to 1 (0.989), suggesting very strong temporal autocorrelation in the error process between NHS Digital data and the CDW. The model was repeated with ρ_T fixed at 1 which represents perfect temporal auotcorrelation. The Deviance Information Criterion (DIC), an indicator of model fit for hierarchical models, was equivalent (DIC=-7299) for the models with fixed and variable temporal dependency. Hence for sake of parsimony, we proceeded with the temporal parameter fixed at 1.

2.4.1 Model output

Table S3 shows the results of the model fit.

Table S3: Median parameter estimates, 95% credible intervals (CI), and effective sample size (ESS) for the spatio-temporal GP-registration model based on 2,000 independent MCMC samples.

Parameter	Coefficient	95% CI	ESS
ρ_s	0.353	(0.264, 0.450)	2000
σ^2	0.000173	(0.000144, 0.000207)	2138
τ^2	0.00263	(0.00226, 0.00303)	2000
β_0 (Intercept)	6.111	(6.088, 6.136)	1360
β_1 (log(CDW))	0.000839	(0.000817, 0.000860)	2000
β_2 (Year)	-0.0152	(-0.0173, -0.0130)	1535
β_3 (Missing GPs)	1.302	(1.203, 1.399)	2000

Figure S1 is a boxplot of the LSOA-level true GP-registered population; observed years are shaded in grey and the predicted years shaded in green. The predicted years are very similar to that

of 2014. This is supported by census data over the same time period which shows a plateau in the total adult population between 2012-2014 (Figure S2). Although the census population is not identical to the GP-registered population, it is still a good indicator of overall trends [5].

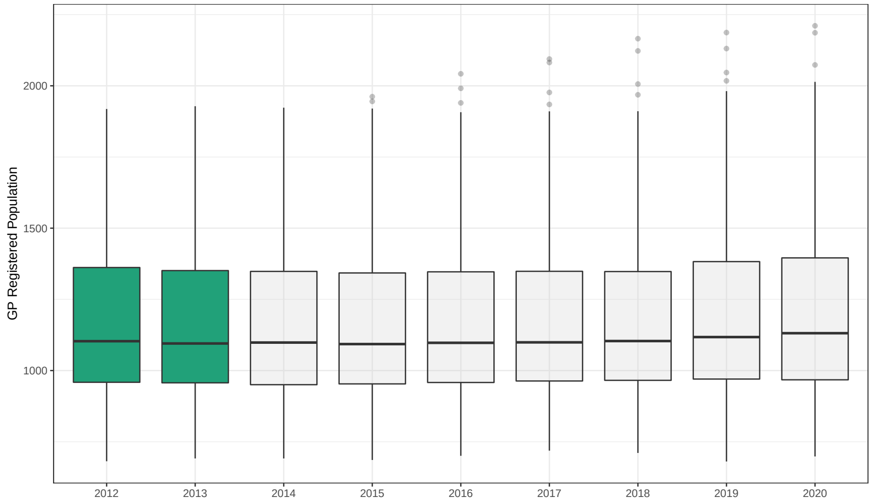

Figure S1: Spread of LSOA-level GP-registered adult (≥25) population from NHS Digital data for years 2014-2020 (shown in grey) and spatio-temporal model prediction results for years 2012-2013 (shown in green).

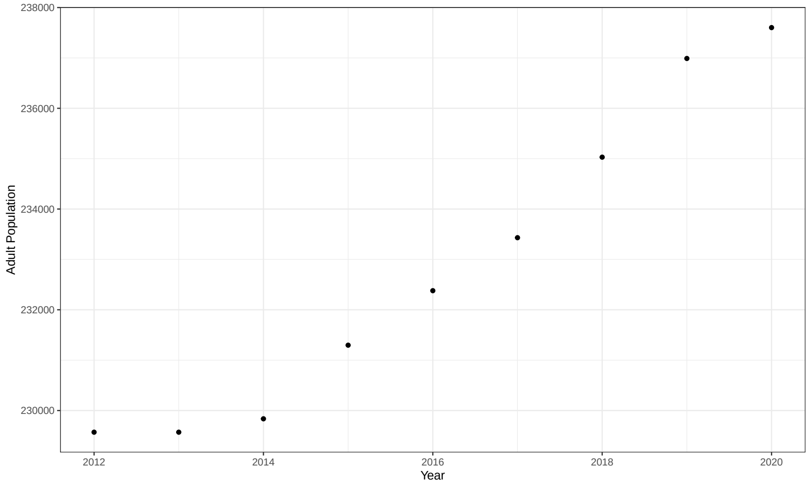

Figure S2: Total population size for the 204 study LSOAs according to ONS mid-year estimates.

The predictive performance of the spatio-temporal model was by leave-one-out cross validation on the observed data (2014-2020). The mean absolute percentage error (MAPE) was calculated for the predictions as well as the percentage of LSOA-level true values within the 95% credible intervals for the corresponding prediction (Figure S4). The model predicts well for years 2014-2019, with a maximum of three LSOAs falling outside the 95% CI. The year 2020 performs considerably worse compared to the others, likely due to impact of the COVID-19 lockdown on movement of people and GP registration behaviour.

Table S4: Mean absolute percentage error (MAPE) and proportion of true values that are within the 95% credible interval (CI) for each year predicted.

**Year predicted**	**MAPE**	**LSOAs in 95% CI**
2014	1.81	202 (99.0%)
2015	1.14	202 (99.0%)
2016	1.21	204 (100.0%)
2017	1.02	204 (100.0%)
2018	1.11	201 (98.5%)
2019	1.36	202 (99.0%)
2020	3.28	168 (82.4%)

2.4.2 MCMC diagnostics

Figures S3-S5 show the traceplots and density curves for the parameters in the model and a subset of the latent variables. The ESS for the model parameters can be seen in Table S3, and the ESS for the latent variables had a median of 2,000 and a minimum of 1,104.

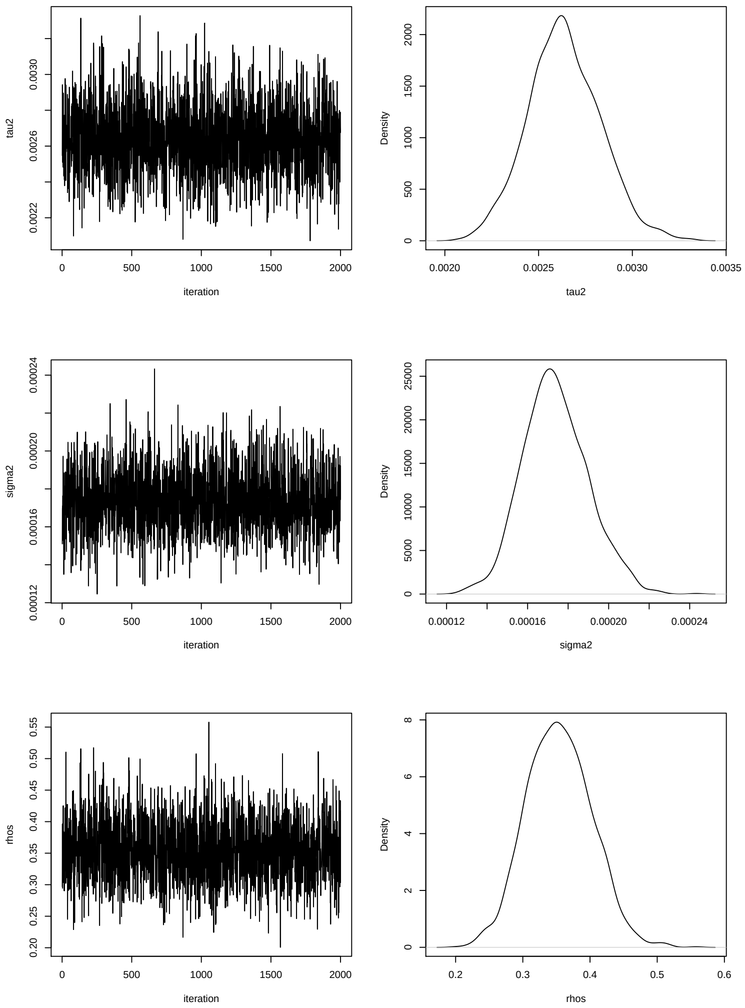

Figure S3: Diagnostic traceplots and density curves for $(\tau^2, \sigma^2, \rho_s)$ in the spatio-temporal GP registration model.

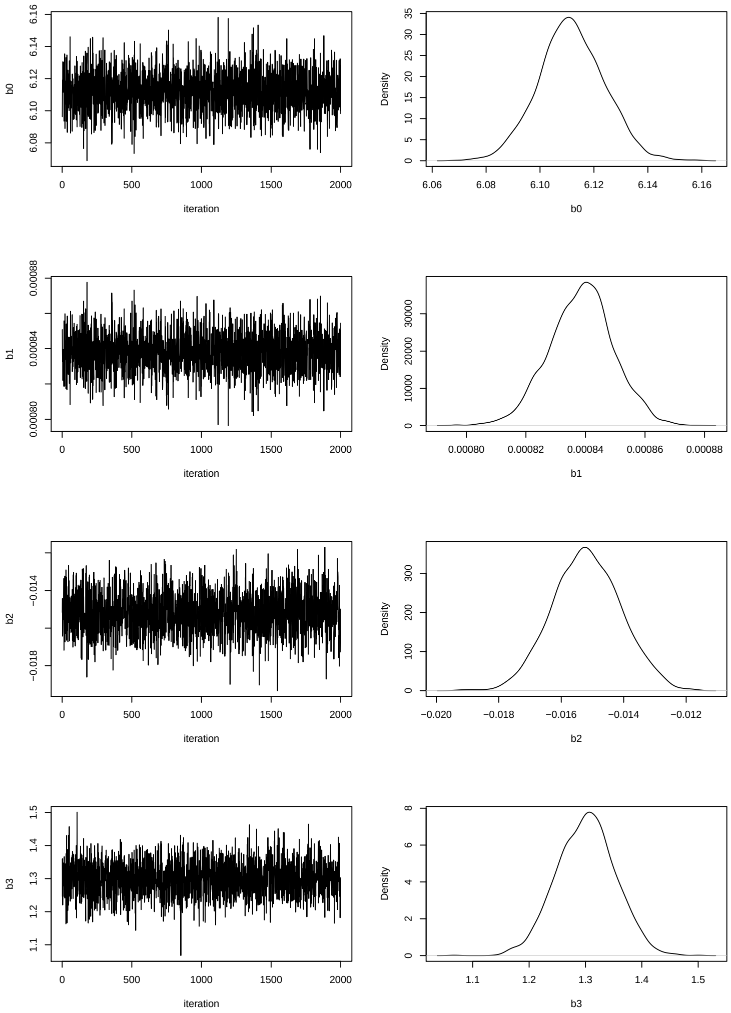

Figure S4: Diagnostic traceplots and density curves for the $\boldsymbol{\beta}$ parameters in the spatio-temporal GP registration model.

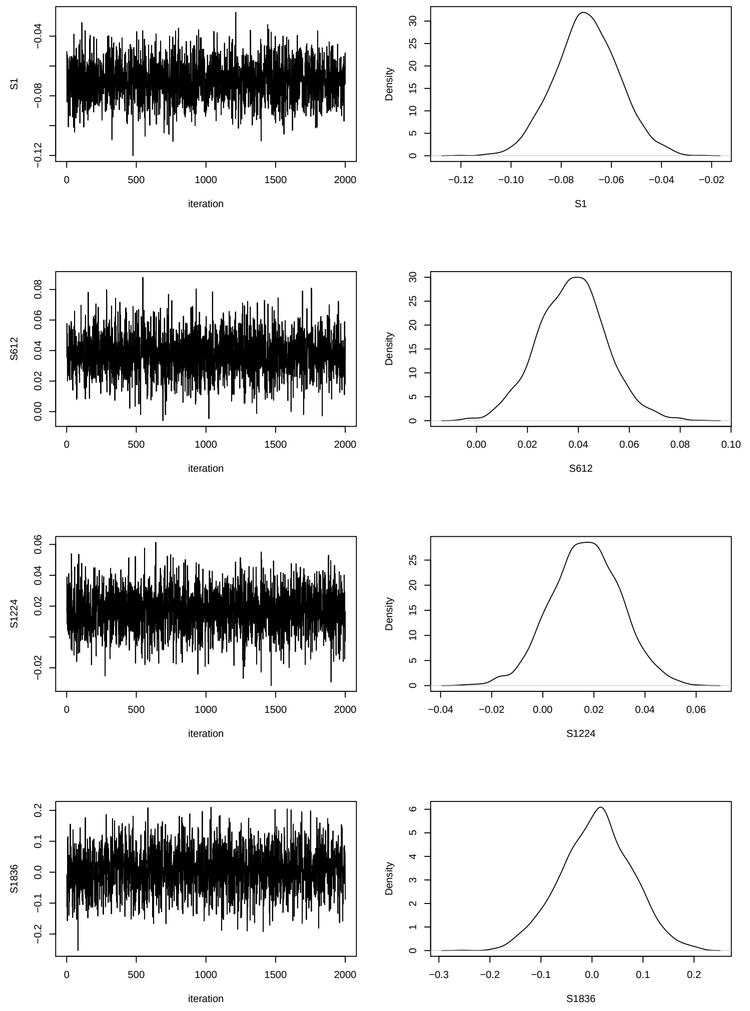

Figure S5: Diagnostic traceplots and density curves for a subset of the $\boldsymbol{S}$ latent variables in the spatio-temporal GP registration model.

3 Outpatient referrals model

This section supplements the main article by providing additional details of the generalised linear mixed model (GLMM) for outpatient referrals. We first present exploratory data analysis results before describing the specifics of the MCMC algorithm used for model fit. The material is assumed to be read in conjunction with the main article.

3.1 Exploratory data analysis

Table S5 provides a summary of the variables used in the referrals model, including data source, general description, and additional notes.

Response variable

Figure S6 shows the time trend in the raw referral counts data by MBRN intervention status. For the sake of this figure, we dichotomiose the MBRN covariate so that an LSOA is classed as "MBRN" if the proportion of the population registered at an MBRN GP $\geq 50\%$ and "Non-MBRN" otherwise. Prior to the initiation of the MBRN, the time trends are quite similar between the two groups. Post-initiation, the MBRN areas show a dramatic decrease in annual number of referrals whilst non-MBRN areas continue in an upward trend. However, the patterns in this plot do not account for population growth or changes in the demographic or health structure of the populations.

Explanatory variables

Table S6 provides summary statistics of the covariates used in the final model for referrals to outpatient respiratory clinics.

Table S6: Summary of model covariates over all space-time units. All values are percentages, except the distance variable which is in kilometres.

	**Min**	**1st quartile**	**Median**	**Mean**	**3rd quartile**	**Max**
65-74	4.40	13.73	16.42	16.88	20.52	32.70
75+	2.33	9.90	13.54	14.09	16.79	34.71
Male	40.19	46.63	47.88	48.20	49.43	65.66
Distance	0.87	3.31	6.81	9.42	13.27	38.16
IMD	3.62	10.84	16.72	22.62	29.19	77.84
MBRN	0	0.20	51.46	50.93	99.90	100.00

Table S7 shows the change in mean of all time-varying covariates: both age variables show a mostly increasing trend, indicative of an ageing population and percentage male has increased marginally. The proportion of the population registered at a GP that joined the MBRN in 2017 remained

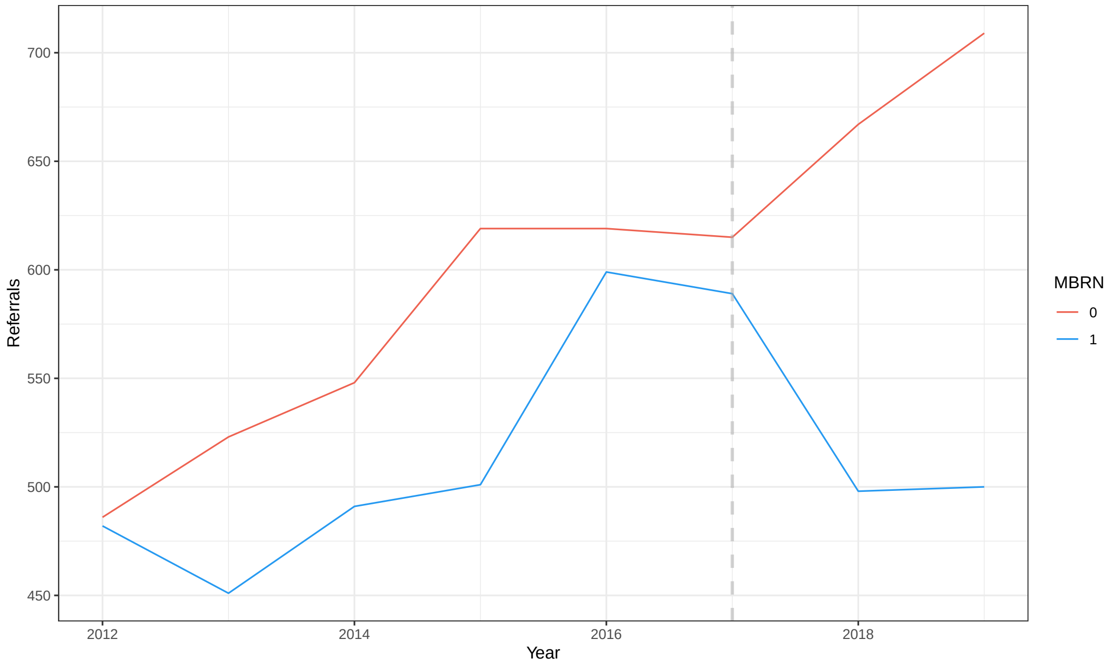

Figure S6: Annual number of referrals for intervention and non-intervention areas. The MBRN covariate has been dichotomised at the 50% mark. The grey dashed line represents the introduction of the MBRN in 2017.

Table S5: Description of variables used in the random intercept model of referrals to outpatient respiratory clinics.

**Variable**	**Time varying (Y/N)**	**Source**	**Description**	**Notes**
Outpatient referrals	Y	CDW (secondary care records)	Annualised count of number of referrals to outpatient respiratory clinics	New referrals from GP, for adults aged 25+ years residing within MBCCG boundaries.<br>Clinic inclusion: respiratory, spirometry, lung, or oxygen clinics; nurse or consultant led; at Royal Lancaster Infirmary, Furness General Hospital, or Westmorland General Hospital.<br>Clinic exclusion: post-op, rheumatology, physio, asthma biologics, or sleep clinics, and 2-week-wait cancer referrals.
CRD patients	Y	NHS Digital and spatio-temporal model output	Annualised count of number of patients with an asthma, COPD, bronchiectasis, or ILD diagnosis	Patients identified by relevant asthma, COPD, bronchiectasis, and ILD SNOMED CT codes. Additional criterion for asthma diagnosis is an inhaler prescription in the last 12 months.
Age	Y	ONS (mid-year estimates)	Percentage of adult (25+ years) population in a given age bracket.	Age brackets '65-74' and '75+' are used; covariate selection methods suggests these are the only relevant age groups.
Sex	Y	ONS (mid-year estimates)	Percentage of adult (25+ years) population that are male	
IMD score	Y	Ministry of Housing, Communities & Local Government	Index of Multiple Deprivation (IMD) score from English Indices of Deprivation	The IMD is update every 3-4 years. The mean of the 2015 and 2019, the two publications within the study period, indices was taken.

Distance to hospital	N	OSMR	Travel distance (km) by car to the nearest hospital within the MBCCG	Hospitals considered: Royal Lancaster Infirmary, Furness General Hospital, and Westmorland General Hospital.<br>Distances were calculated using open source routing software in R Studio. Distances were calculated for all 11,594 (as of 14/01/22) postcodes in the study area and then averaged by LSOA.
Year	Y	NA	Factor variable form of year	Factor form used as opposed to continuous to better study the evolution of the MBRN in the three years since initiation.
MBRN intervention	Y	NHS Digital	Percentage of GP-registered population registered at an MBRN GP	This is calculated for each year regardless of whether the MBRN was yet active in order to account for baseline differences in the areas that have and have not received MBRN intervention. For study years prior to MBRN introduction (2012-2016), the covariate is calculated as the percentage of the population registered at a GP that goes on to join the MBRN in 2017.<br>Percentage calculated using LSOA-level GP registration data released by NHS Digital. We do with calculations with all-age data and assume this variable not to be correlated with age. As with the 'GP-registered population' variable, mean taken across quarters.<br>For the study years 2012 and 2013, the 2014 value is used. Exploratory analysis suggests numbers registered at each GP does not fluctuate year-on-year.

mostly constant between 2014-2017 before increasing in 2018. MBRN coverage is unobserved for study years 2012 and 2013 as NHS Digital did not release LSOA-level data until 2014. In the final model, we assume the 2012 and 2013 values to be equal to 2014. Note that IMD is not included in Table S7 since it is not time varying.

Table S7: Mean of time varying GLMM covariates by study year.

**Year**	**65-74**	**75+**	**Male**	**MBRN**
2012	16.0	13.5	48.0	-
2013	16.5	13.7	48.0	-
2014	16.8	13.9	48.0	50.7
2015	17.0	14.0	48.3	50.7
2016	17.2	14.1	48.3	50.8
2017	17.3	14.2	48.3	50.8
2018	17.2	14.5	48.4	51.4
2019	17.1	14.8	48.4	51.6

Figures S7 and S8 both illustrate the spread of the MBRN. Since the MBRN covariate is time-varying we used 2017 data only for these plots. The MBRN covariate is defined as percentage of the total population (Table S6), yet 84% of the data points are either less than 1% or greater than 99%, with an overall median of 51.5% (S7). Lancaster is the only area that has had widespread full coverage (Figure S8), this is because all GPs in this area are part of larger, multi-site practices. The LSOAs with 0% coverage account for a greater proportion of the MBCCG spatially speaking, due to the differences in population density (illustrated by the sizes of the LSOAs), and yet phase 1 of the MBRN reached 50% of the total MBCCG population.

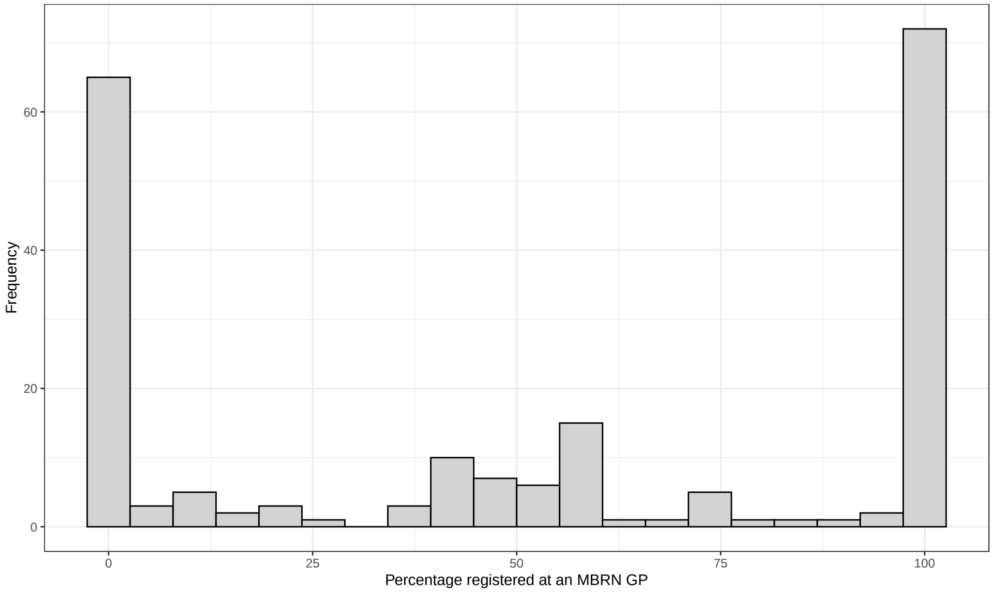

Figure S7: Histogram showing the distribution of the proportion of the popualtion registered at an MBRN GP in 2017.

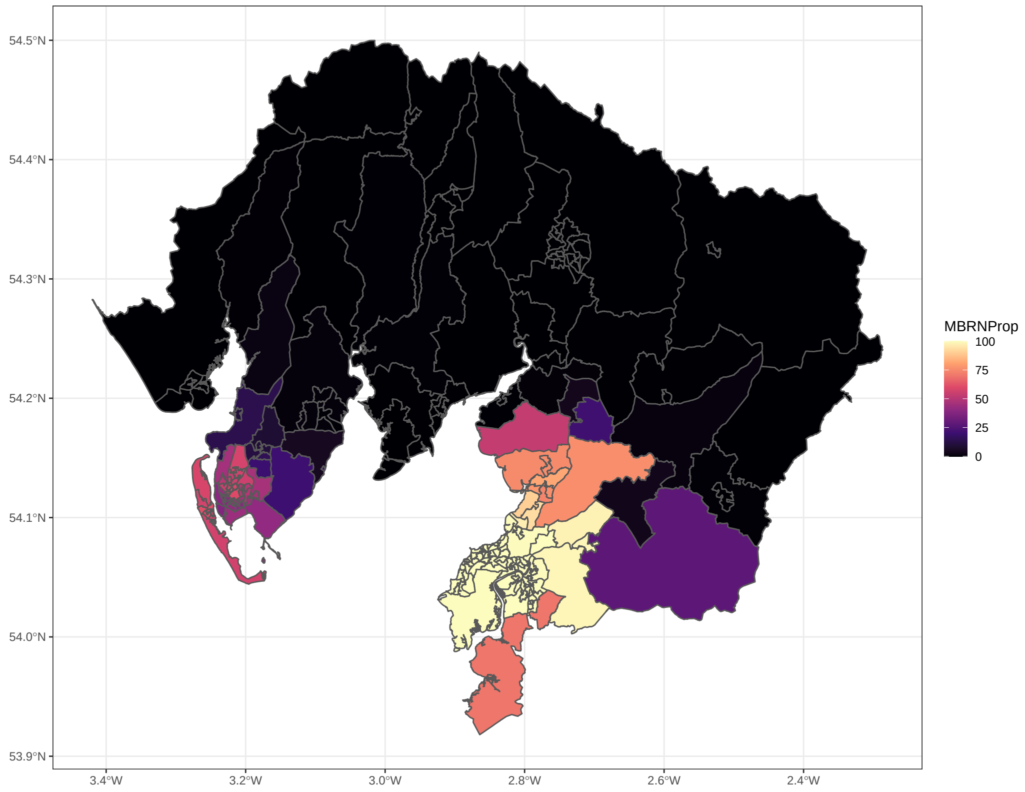

Figure S8: Choropleth map of percentage of LSOA population registered at a GP that joined the MBRN in 2017.

Overdispersion

We first considered a Poisson GLM to model referrals, with covariates included as described in the main article. The model was overdispersed ($\text{mean}(Y_{it}) = 5.5 < 10.0 = \text{var}(Y_{it})$; residual deviance $= 2092 > 1707 = q(0.95, df = 1612)$ and random effects models were next considered. An analysis of the residuals did not suggest any significant spatial or temporal correlation. Moran's I statistic was insignificant for study years 2012-2017 and suggested only a weak spatial correlation at 5% significance level for 2018 and 2019 (Moran's I = 0.10 and 0.15 respectively). Therefore, it was concluded that a more complex spatial correlation structure was not necessary and an independent random intercept model was used.

3.2 MCMC methodology

Prior distributions:

The random effects, Z_i $(i = 1, \ldots, N)$, act as latent variables and have independent $\text{Normal}(0, \kappa^2)$ priors, as described in the main article. For the remaining parameters, the following priors were

used:

$$\kappa^2 \sim \text{Gamma}\,(a,b)$$
$$\gamma \sim \text{N}\left(0, \lambda^2 I\right) \ ,$$

where $a = 1$, $b = 0.01$, $\lambda^2 = 1000$, and I_p is a p-dimensional identity matrix with p being the number of regression parameters in the referrals model.

Posterior distribution:

Let $\theta = (\gamma, Z, \kappa^2)$ be the vector of parameters to be estimated. The joint posterior distribution is:

$$\begin{aligned}\pi\left(\theta|Y\right) \propto \exp &\left(\sum_{i=1}^{N}\sum_{t=1}^{T}\left[y_{it}^T d_{it}\gamma + y_{it}^T z_i - \exp(\log(R_{it}) + d_{it}\gamma + z_i)\right]\right) \\ &\times (\kappa^2)^{-N/2}\exp\left(-\frac{1}{2\kappa^2}\sum_{i=1}^{N} z_i^2\right) \\ &\times \pi(\kappa^2,\gamma) \ ,\end{aligned}$$

where $\pi(\kappa^2, \gamma)$ represents the corresponding prior distributions.

Updating algorithms:

The parameter κ^2 was updated via a Gibbs sampler according to the following full conditional posterior distribution:

$$\kappa^2|Y, \theta_{-\kappa^2} \sim \text{Inverse-Gamma}\left(a + \frac{N}{2}, b + \frac{1}{2}\sum_{i=1}^{N} z_i^2\right) \ .$$

The regression parameters, γ, and latent variables, Z, were updated jointly. If we let $\psi = (\gamma, Z)$, the posterior distribution for ψ is:

$$\pi(\psi|Y,\kappa^2) \propto \exp\left(-\frac{1}{2}\psi^T Q\psi + Y^T B\psi - \mathbf{1}^T\exp(\log(R) + B\psi)\right) \ , \tag{2}$$

where Q is a diagonal matrix of the prior precision and B is the design matrix for ψ. The distribution in (2) does not have a tractable form but can be approximated to a GMRF using the methodology in e.g., Chapter 4 of Rue and Held (2005) [4]. The GMRF approximation, which we will denote by $q(.)$, is then used as a proposal distribution in a Metropolis-Hastings step.

We omit the full calculations, but in brief, the approximation uses a second-order Taylor's expansion of the log of the posterior in (2) about the current value of ψ, say ψ_0. Then,

$$\pi(\psi|Y,\kappa^2) \approx q(\psi|Y,\kappa^2,\psi_0) \propto \exp\left(-\frac{1}{2}\psi^T C\psi + b\psi\right) \sim N\left(C^{-1}b, C^{-1}\right) \ ,$$

where the matrix C and vector b are functions of ψ_0:

$$b = \left(\log \pi(\psi_0|Y,\kappa^2)\right)' - \left(\log \pi(\psi_0|Y,\kappa^2)\right)'' \psi_0$$
$$C = -\left(\log \pi(\psi_0|Y,\kappa^2)\right)'' \ .$$

To improve the accuracy of the approximation, the expansion is repeated, with each successive expansion performed around the mean of the previous approximation i.e. the first expansion is around ψ_0, the second expansion is around $\mu_1 = C^{-1}(\psi_0)b(\psi_0)$, the third expansion is around $\mu_2 = C^{-1}(\mu_1)b(\mu_1)$, and so on. Preliminary runs of the algorithm found five expansions to be sufficient. Once the approximation is complete, a proposed value, say ψ^*, can be sampled from the GMRF according to the same methodology in 2.3.2. As with the spatio-temporal GP registration model, we impose the linear constraint $\sum_i \psi_i = 0$ to ensure the precision matrix of the GMRF is invertible.

The acceptance probability of the Metropolis-Hastings step is,

$$\alpha = \min\left\{1, \frac{\pi(\psi^*|Y,\kappa^2)q(\psi_0|\psi^*)}{\pi(\psi_0|Y,\kappa^2)q(\psi^*|\psi_0)}\right\} \ .$$

Note that the above also requires a Taylor's expansion of $\pi(\psi^*|Y,\kappa^2)$ around ψ^* in order to evaluate $q(\psi_0|\psi^*)$. This is also iterated to improve the accuracy.

Finally, at each iteration of the MCMC algorithm, we randomly sample from the posterior predictions of P_{it}^{NHS} for study years 2012 and 2013, and update the offset term according to the correction formula in Methods: Statistical Analysis: *1. Adjusting CRD patient count* of the main article. Using this method, we use the entire posterior predictive sample, as opposed to a point estimate such as the mean of the sample, and thus account for the uncertainty in the predictions.

Inference

Inference was based on 2,000 independent samples obtained from 25,000 iterations of the algorithm, with a burn-in of 5,000 and the remaining 20,000 thinned by a factor of 10. Similarly to the registration model, convergence was established by the Gelman-Rubin convergence diagnostic. Trace plots, density curves, auto-correlation plots, and ESS calculations were used to assess sufficient mixing of the chain. Continuous explanatory variables were standardised prior to model fit to reduce multicollinearity.

3.3 MCMC Diagnostics

Figures S9-S11 show the traceplots and density curves for the parameters in the model. Since there are 21 regression coefficients and 204 latent variables, only a subset of the plots are displayed. The ESS for κ^2 was 1,432. The ESS for the regression coefficients had a median of 2,000 and a minimum of 1,863, and the latent variables had a median of 2,000 and a minimum of 1,231.

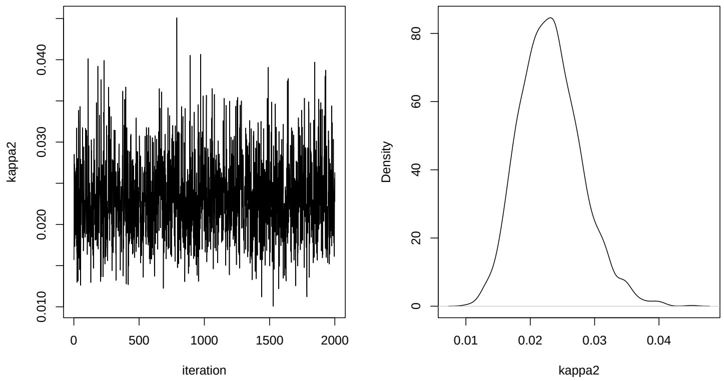

Figure S9: Diagnostic traceplots and density curves for κ^2 in the random intercept outpatient referrals model.

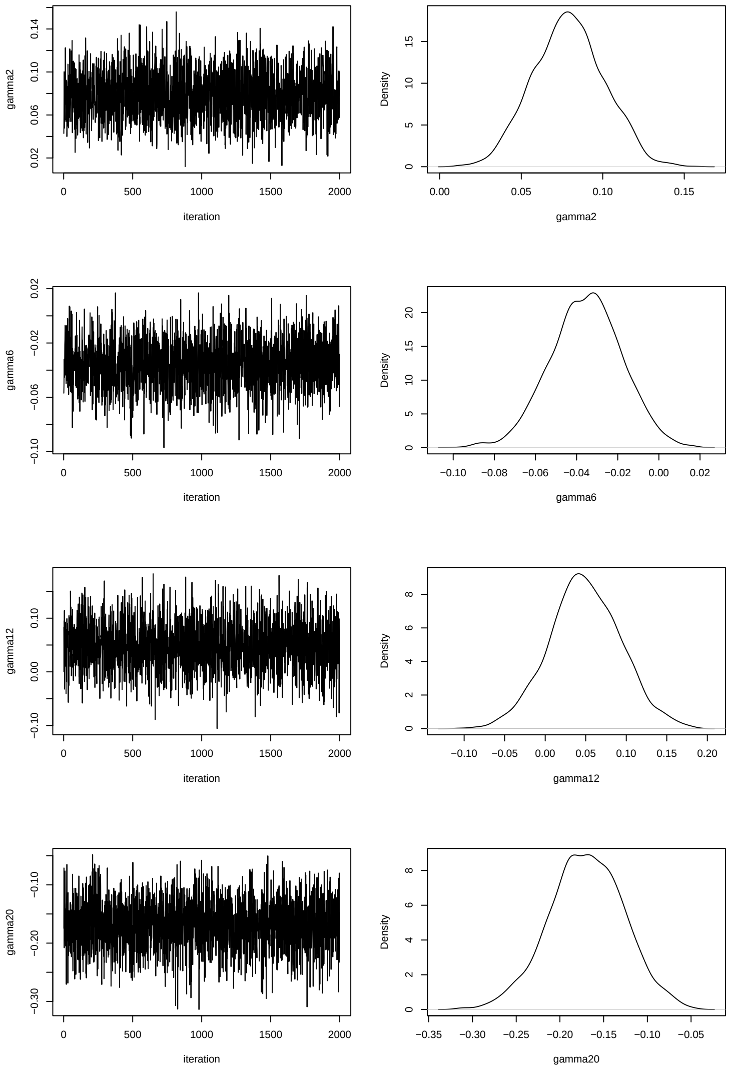

Figure S10: Diagnostic traceplots and density curves for a subset of the regression coefficients, $\boldsymbol{\gamma}$, in the random intercept outpatient referrals model.

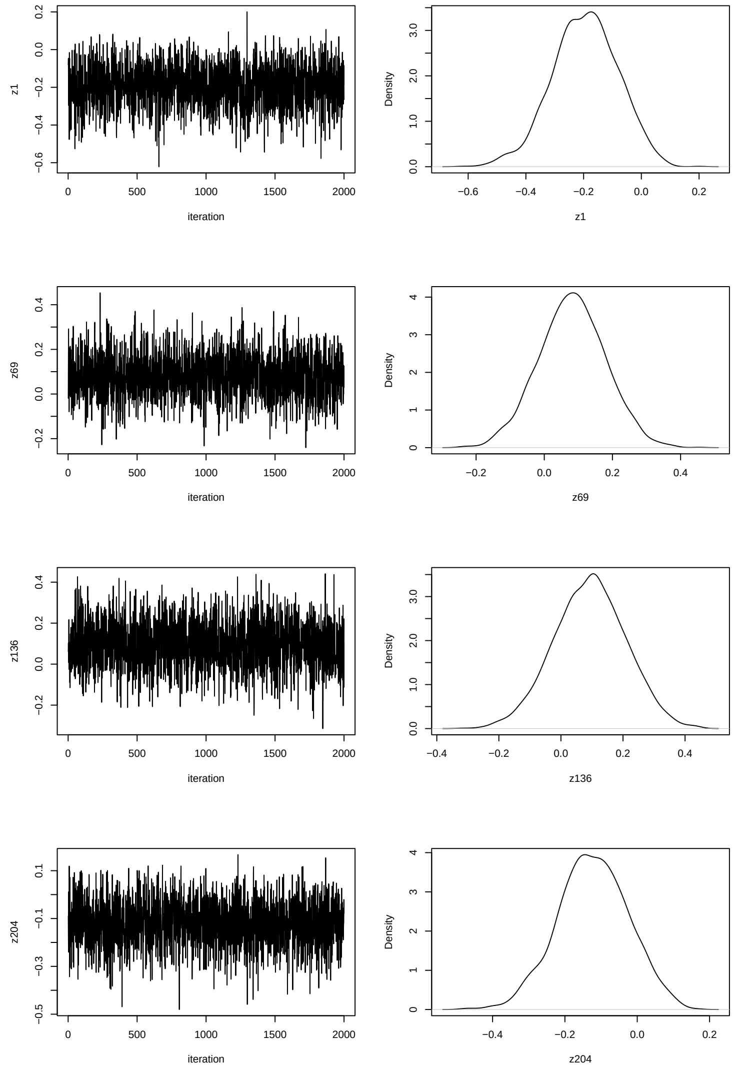

Figure S11: Diagnostic traceplots and density curves for a subset of the $\boldsymbol{Z}$ latent variables in the random intercept outpatient referrals model.

References

[1] Rushworth, A., Lee, D., and Mitchell, R. (2014) A spatio-temporal model for estimating the long-term effects of air pollution on respiratory hospital admissions in Greater London. *Spatial and Spatio-temporal Epidemiology,* **10**, 29–38.

[2] Leroux B.G., Lei X., B. N. (2000) Estimation of Disease Rates in Small Areas: A new Mixed Model for Spatial Dependence. In Halloran M.E., B. D., (ed.), *Statistical Models in Epidemiology, the Environment, and Clinical Trials*, pp. 179–191 Springer New York.

[3] Lee, D. (2011) A comparison of conditional autoregressive models used in Bayesian disease mapping. *Spatial and Spatio-temporal Epidemiology,* **2**, 79–89.

[4] Rue, H. and Held, L. (2005) Gaussian Markov random fields theory and applications, Monographs on statistics and applied probability ; 104Chapman & Hall/CRC, Boca Raton.

[5] Office for National Statistic Beyond 2011: Administrative Data Sources Report: NHS Patient Register. (2012).
